# A new sonographic marker in the diagnosis of prenatal bilateral renal agenesis, segmental anterior deviation of the aorta

**DOI:** 10.1515/crpm-2022-0001

**Published:** 2022-07-12

**Authors:** Halis Özdemir, Belma Gözde Özdemir, Songül Yerlikaya Kavak, Şule Şık

**Affiliations:** Department of Obstetrics, Gynecology and Reproductive Sciences, Division of Perinatology, Malatya Turgut Özal University Training Research Hospital, Malatya, Turkey; Department of Pathology, Malatya Turgut Özal University Training Research Hospital, Malatya, Turkey

**Keywords:** congenital anomaly, renal agenesis, segmental aortic deviation, ultrasonography

## Abstract

**Objectives:**

Bilateral renal agenesis is a rare congenital anomaly that is associated with high neonatal mortality. Bilateral renal agenesis is most often present with anhydramniosis in the mid-trimester.

**Case presentation:**

We report a case of bilateral renal agenesis diagnosed prenatally. We presented the ultrasound and pathology images of this fetus with a new sonographic sign, segmental anterior deviation in the abdominal aorta.

**Conclusions:**

To our knowledge, this is the first reported case of a fetus with a segmental aortic anterior deviation.

## Introduction

Bilateral renal agenesis is characterized by the complete absence of kidneys and related constitutes. Bilateral renal agenesis occurs in 1 to 3 per 10,000 pregnancies, and is incompatible with life [1]. Bilateral renal agenesis cases often present with anhydramniosis in the mid-trimester. A basic combination is an empty renal fossa and an empty fetal bladder [1]. In addition, failure of Doppler imaging of the renal artery and “downstream” adrenaline are other findings to strengthen the diagnosis [2].

However, when the fetal adrenal gland fills the entire renal fossa due to its inferior location, this can lead to misdiagnosis [3]. The sonographer might not adequately and appropriately display the renal fossa. Situations that can prevent this include oligohydramnios, maternal obesity, or fetal position. Therefore, it is crucial to evaluate carefully the renal fossa to avoid mistakes.

Bilateral renal agenesis is associated with high neonatal mortality and poor prognosis. This syndrome is associated with severe oligohydramnios and pulmonary hypoplasia, and these conditions are generally incompatible with life. In addition to these sonographic findings mentioned above, in this case report, we present the ultrasound and pathology images of a fetus with bilateral renal agenesis with segmental deviation in the abdominal aorta.

## Case presentation

**Figure 1:**
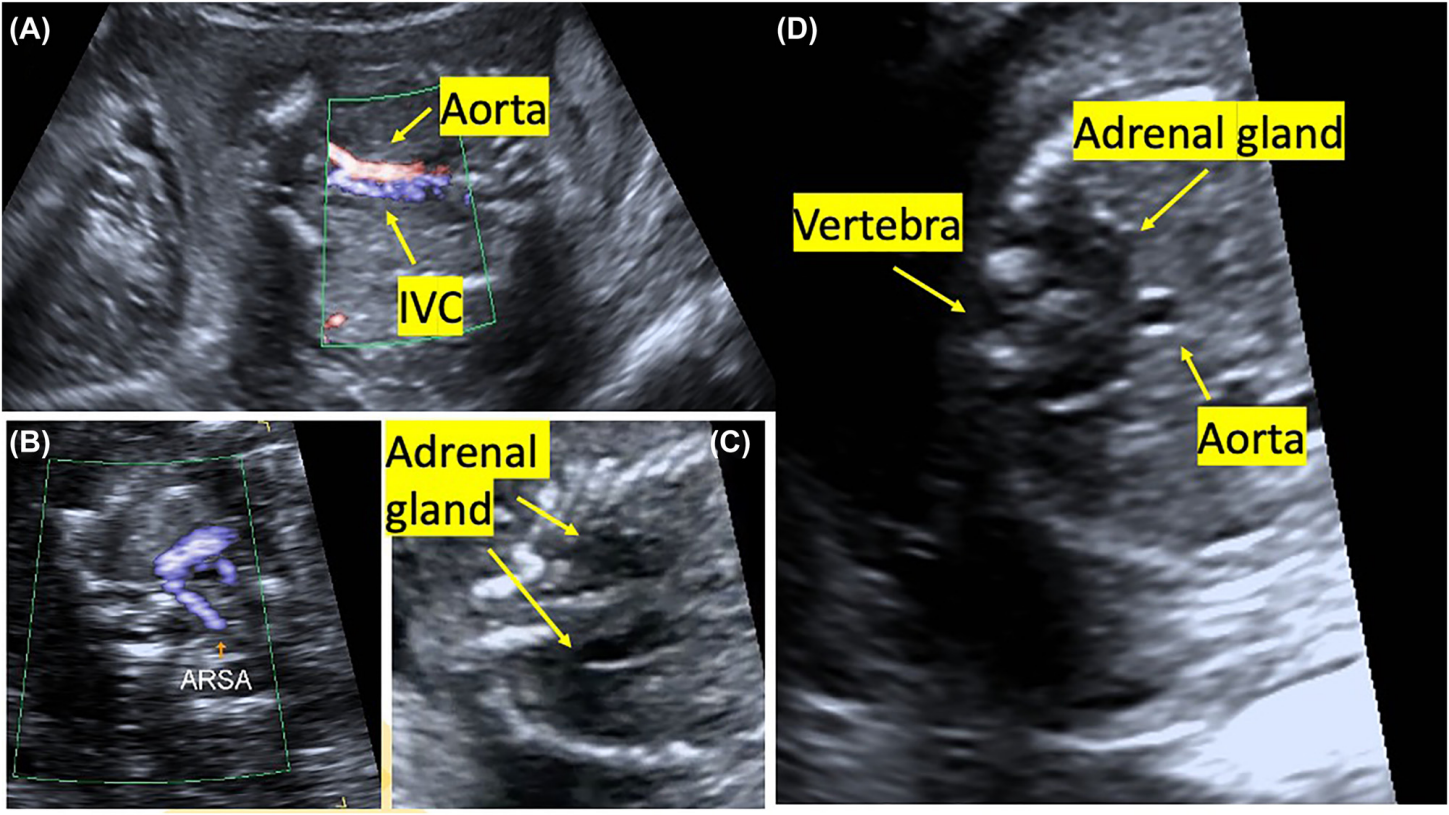
Sonographic images of an 18-weeks-old fetus with bilateral renal agenesis. (A) Fetal abdominal aorta, renal arteries are not observed (IVC: inferior vena cava). (B) Aberrant right subclavian artery (ARSA). (C) Downstream linear adrenal gland. (D) In the transverse section, fetal adrenal glands are prominent and extend towards the back of the aorta.

Our patient is an 18-years-old woman referred to our clinic because of anhydramniosis during her first pregnancy. The gestational week was calculated as 18 weeks and 1 day, according to the last menstrual period. In the detailed fetal sonography, bilateral renal fossa and the bladder were observed empty with anhydramnios. Bilateral renal arteries could not be observed on color Doppler imaging (Figure 1A). Aberrant right subclavian artery (ARSA) was present in fetal cardiac evaluation (Figure 1B). Adrenal glands were clearly observed and linear (Figure 1C). In the transverse section, it was observed that the adrenal glands were prominent and converged behind the aorta (Figure 1D).

**Figure 2:**
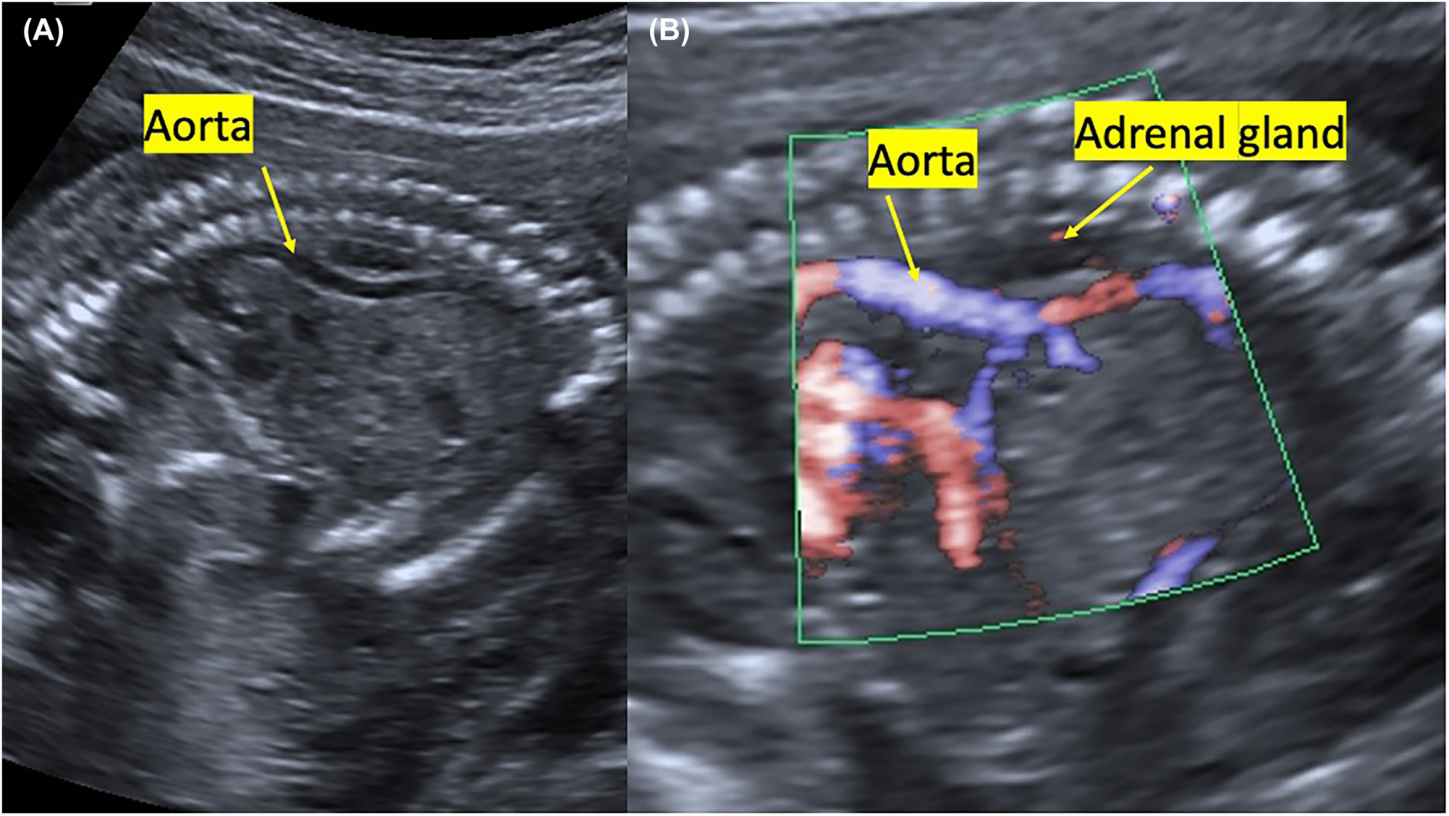
Sagittal view. (A) Deviation anterior to the abdominal aorta at T11-L1 level. (B) Color Doppler image.

**Figure 3:**
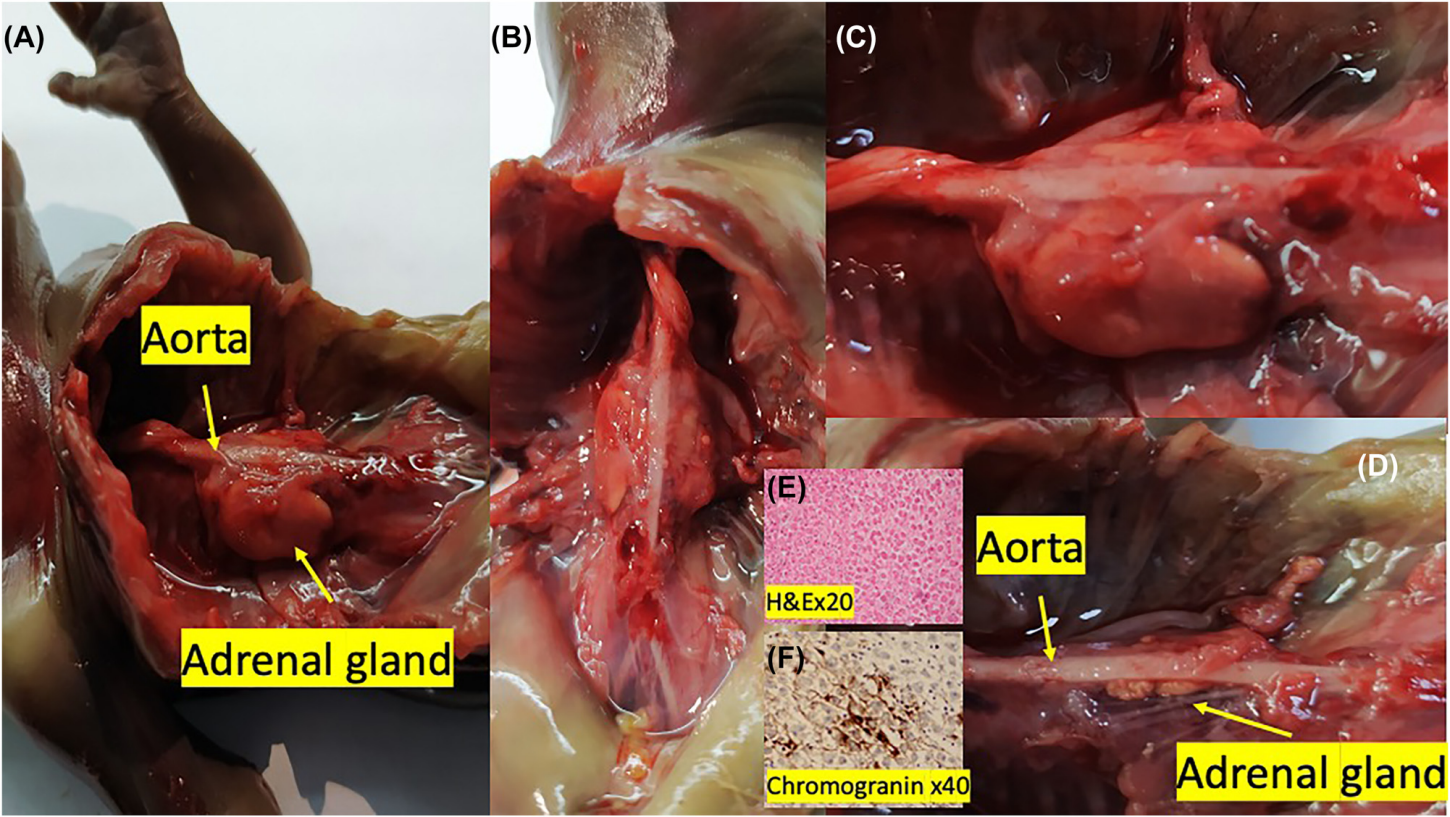
Fetal autopsy. (A, B, C) Appearance of adrenal glands in the renal fossa. (D) View after excision of the right adrenal gland, view of the left adrenal gland posterior to the fetal aorta. (E) Staining with hematoxylin-eosin (H&E) ×20. (F) chromogranin staining ×40.

Also, by sagittal imaging, we observed segmental anterior deviation in the aorta between T11 and L1 in the fetal abdominal aorta, at the level where the celiac trunk originated (Figure 2A, B). Fetal biometry was observed compatible with 18 weeks. After termination, at autopsy of the fetus, the kidneys were not found in the bilateral renal fossa (Figure 3A, B, C). It was observed that the structure, which was thought to be the adrenal gland macroscopically, filled the renal fossa in a rectangular shape. It was observed that both adrenal glands’ upper ends extended towards the back of the aorta (Figure 3D). It was confirmed by hematoxylin-eosin and immunohistochemical staining that this structure was the adrenal gland (Figure 3E, F). Placenta and fetal skin biopsy were done for genetic analysis. The subsequent cytogenetic and molecular karyotypes were reported as normal.

## Discussion

Bilateral renal agenesis can be diagnosed prenatally. The diagnostic criteria are oligohydramnios and the inability to show fetal kidneys and bladder on ultrasound [4]. Additional sonographic markers defined in the literature are a lack of monitoring of renal arteries with Doppler and linear downstream monitoring of the adrenal gland [1]. In this case report, in addition to the above criteria, we observed anterior segmental deviation of the aorta at the upper adrenal gland level on sonography. To our knowledge, this finding has not been previously described in the literature.

Prenatal ultrasonography is the primary imaging model for fetal urogenital evaluation. The earliest kidneys can be monitored by ultrasound in the 9th week of pregnancy. Eighty percent of the fetal kidneys in the 11th week and 92% in the 13th week can be seen ultrasonographically (USG) [4]. Fetal urine production starts between the 8th and 10th week of pregnancy. Although it starts before the 16th week, its major contribution to amniotic fluid is after the 16th gestational week [5]. Therefore, anhydramnios in fetuses with bilateral renal agenesis is a strong marker only after the 16th gestational week. Due to the echogenic appearance of renal structures in the early gestational week, it can often be challenging to separate them from surrounding tissues. The adrenal gland also extends downwards in a rectangular shape, sometimes causing it to be mistakenly described as a kidney [4]. A study reported that the sensitivity of USG in patients with bilateral renal agenesia with postpartum confirmation was 83.7% [6]. In another study, magnetic resonance imaging (MRI) diagnosis showed a slight increase compared to USG (89.5% vs. 85.0%) [7].

The adrenal gland is a structure consisting of the cortex and medulla located above the bilateral kidney. The cortex and medulla have distinct cell origins. A group of cells differentiating from the intermediate mesoderm in the 4th stage of pregnancy forms bilateral adrenogonadal primordium (AGP). Later, a cephalic cell group moves dorsomedially to create the adrenal primordium (AP). This structure is located on the ventral side of the dorsal aorta. On the 7th week of pregnancy, a group of cells originating from the neural crest moves towards the AP area. This structure will later differentiate as the adrenal medulla. Simultaneously, mesenchymal cells in the Bowman’s capsule area migrate to form a fibrous capsule around the developing adrenal. This process ends in the 9th week of pregnancy [8]. The presence or absence of kidneys in the renal fossa affects only the shape of the adrenal gland. Apart from that, the development of the kidney and adrenal gland proceeds utterly independent of each other. The standard sonographic form of the adrenal gland is an inverted Y or V shape, whereas in patients with renal agenesis or ectopia, a linear configuration occurs [9].

**Figure 4:**
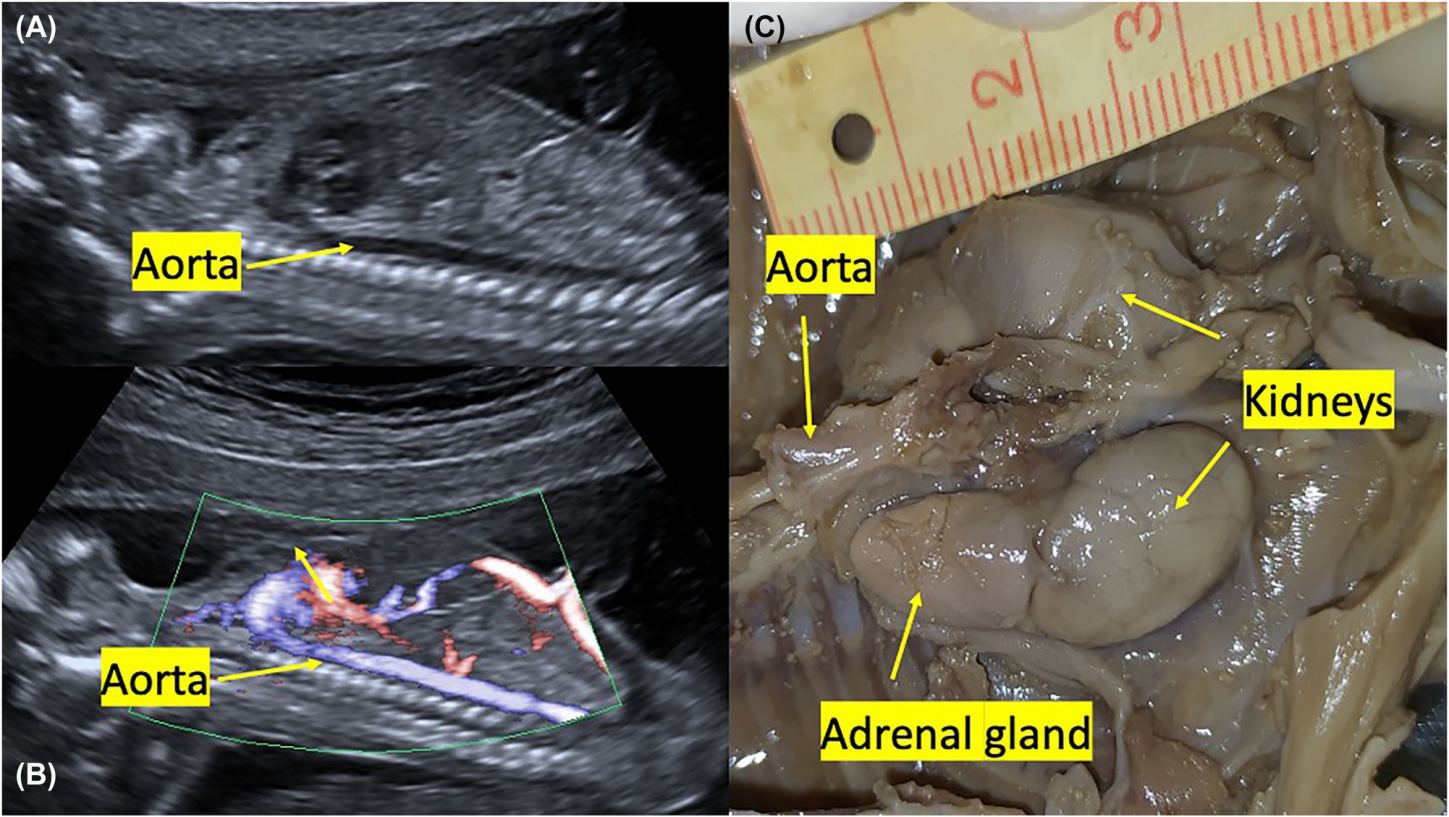
Images of an 18-week normal fetus. (A, B) 2D and color doppler image of the fetal aorta. (C) Fetal autopsy, view of normal kidney and adrenal glands.

In this case report, the fetus had a segmental aortic anterior deviation, a finding that has not been previously noted in the literature. The reason is that, as we have seen in prenatal ultrasound and fetal autopsy, the two adrenal glands are close to each other. The adrenal glands come close to each other in the superior of the gland and push the fetal aorta forward. In normal embryos, both adrenal glands are independently placed in the aorta’s ventrolateral and descend into the aorta. However, in this case, it is accompanied by abnormal migration, and the adrenal glands were located close to each other on the superior side. Figure 4A, B show 2D and Doppler sonography images, including a normal fetus in the abdominal aorta. Figure 4C shows a normal adrenal gland and kidney autopsy image of a fetus terminated due to neural tube defects at the same gestational age and without urinary system anomalies. When we pay attention to the adrenal gland image belonging to the fetus, which is typical for urogenital evaluation in Figure 4C, we can easily see that the adrenal glands are far enough from each other in the superior view. It was observed that there is a space between the adrenal gland and the aorta. However, from Figure 3D, when viewing the opposite side’s adrenal gland from the back of the aorta, we cannot see that gap we see in normal fetuses here.

This case report contributes to the literature by describing an additional sonographic marker that might be found in a fetus with bilateral renal agenesis. Further prospective studies are needed to determine whether this finding is specific for renal agenesis.
